# Recombinase Polymerase Amplification (RPA) Combined with Lateral Flow Immunoassay for Rapid Detection of *Salmonella* in Food

**DOI:** 10.3390/foods9010027

**Published:** 2019-12-26

**Authors:** Jiali Li, Biao Ma, Jiehong Fang, Antong Zhi, Erjing Chen, Ying Xu, Xiaoping Yu, Chuanxin Sun, Mingzhou Zhang

**Affiliations:** 1 Zhejiang Provincial Key Laboratory of Biometrology and Inspection & Quarantine, China Jiliang University, Hangzhou 310018, China; s1709071012@cjlu.edu.cn (J.L.); 16a0701109@cjlu.edu.cn (B.M.); FangJH@cjlu.edu.cn (J.F.); s1409071034@cjlu.edu.cn (A.Z.); s1709071002@cjlu.edu.cn (E.C.); s1809071028@cjlu.edu.cn (Y.X.); yxp@cjli.edu.cn (X.Y.); 2 Department of Plant Biology, Uppsala BioCenter, Linnean Centre for Plant Biology, Swedish University of Agricultural Science (SLU), P.O. Box 7080, SE-75007 Uppsala, Sweden; Chuanxin.Sun@slu.se

**Keywords:** recombinase polymerase amplification, lateral flow dipstick, *Salmonella*, rapid and quantitative detection

## Abstract

*Salmonella* can cause serious foodborne diseases. We have developed a lateral flow immunoassay combined with recombinase polymerase amplification (LFD-RPA) for detection of *Salmonella* in food. The conserved fragment (*fimY*) was selected as the target gene. Under an optimal condition (37 °C, 10 min), the sensitivity was 12 colony-forming units (CFU)/mL in a pure culture. Testing with 16 non-*Salmonella* strains as controls revealed that LFD-RPA was specific to the *fimY* gene of *Salmonella*. The established assay could detect *Salmonella* at concentrations as low as 1.29 × 10^2^ CFU/mL in artificially contaminated samples. This detection was at a slightly higher level than that for a pure bacterial culture. Combined with the test strip reader, the LFD-RPA is a feasible method for quantitative detection of *Salmonella* based on the test line intensity, which was the ratio for the test line and control line of the reflected light. The method could be a potential point-of-care test in limited resource areas and provides a new approach and technical support for the diagnosis of food safety.

## 1. Introduction

With the acceleration of economic globalization and trade liberalization, food safety has been a global public health issue prompting widespread concern [1]. *Salmonella* is an important food-borne pathogen causing food poisoning, which can transmit along the food production chain from farm to an retail chain, leading to severe foodborne diseases [2,3,4]. *Salmonella* is widespread in the natural environment, and can grow under ambient temperature conditions. To date, there are more than 80 million cases of foodborne salmonellosis worldwide [5]. The World Health Organization lists *Salmonella* as a moderately to seriously hazardous foodborne pathogen [6]. *Salmonella* is found in a wide variety of food products, including poultry, vegetables, eggs, and soybean products, and contamination is common in retail meats [7,8]. Accuracy is essential for a detection technique. Development of rapid and quantitative detection is desired for the diagnosis and control of the disease.

The conventional culture method is used as national standard for *Salmonella* detection [9]. The counting based on the method in the inspection and quarantine system has updated in 2016. Although a gold standard, the traditional method is time-consuming and laborious, taking around 5 days to complete the isolation culture and serotyping identification. There are limitations in terms of detection specificity and sensitivity. The complexity of the sample greatly affects not only the biochemical reactions between bacteria in enterobacteriaceae, but also the morphology of the bacterial colony [10]. With the advancements in molecular biotechnology, rapid amplification based on nucleic acid targets is now a common method in the laboratory. For the past few decades, polymerase chain reaction (PCR) has been the most widely used technique for DNA amplification. However, it requires precision temperature-controlled instruments with highly trained staff. The industry standard for import and export has used real-time PCR to detect *Salmonella* [11]. These restrict the application in the field, where there are resource limitations [12].

On the basis of understanding the principle of nucleic acid amplification, isothermal nucleic acid amplification has gradually become an alternative protocol. Combined with the cognition of recombinant technology, the researchers have paid more attention to developing point-of-care and rapid technology. As a new technology of nucleic acid isothermal amplification, recombinase polymerase amplification (RPA) has shown greater advantages in detection for its great sensitivity, low-cost, and high speed, and detection without complex instruments in resource-poor laboratories and the outdoors, which are typically lacking traditional molecular detection methods [13]. This technique mainly locates homologous sequences in double-stranded DNA by combining the recombinase with a protein-DNA complex, which is formed by binding of the primer. The exponential amplification is carried out on the target fragment by initiating a chain exchange reaction to form DNA synthesis [14]. The amplified products can typically be detected after operating only 5–10 min optimally at 37–40 °C. Since the first report in 2006, RPA has been emerging in medical diagnosis [15,16,17], foodborne pathogens [18,19], and genetically modified crops [20,21], as well as research on viruses [22,23]. For the detection of *Salmonella*, the *fimY* gene has been reported as unique to the *Salmonella* species and hence is suitable to use as an appropriate target gene for detecting *Salmonella* [24]. The *fimY* gene is involved in regulating type 1 fimbrial expression, which are the most common fimbriae in the *Salmonella* species [25]. Additionally, the amino acid sequence of *fimY* shares very little homology with other known prokaryotic proteins in the GenBank database [24]. Thus, the *fimY* gene was selected as the target gene in this study.

A portable, compact, homoiothermal instrument was considered for visual inspection of the RPA amplification product. In view of the market prospects, RPA, in combination with a lateral flow dipstick (LFD), is the ideal choice for point-of-care detection in a resource-limited setting [26]. The LFD is a simple device for endpoint testing with an antibody specific to an antigen, in which colloidal gold is commonly used for labelling. If the amplification succeeds, a red line is produced on the test wire due to the accumulation of colloidal gold. In the absence of target DNA, no red band results in a test line. The result is directly observable by the naked eye in a short time. In the present study, we describe the development and application of the LFD-RPA assay aiming to amplify the target fragment of *Salmonella*, which could provide better efficiency and sensitivity. The test strip reader (TSR-100, Allsheng Instruments Co. Ltd., Hangzhou, China) was used to scan strip results. After determining the analytical sensitivity and specificity, we applied this method to actual food samples. By comparison with the real-time quantitative PCR (TaqMan probe) method, we conclude that the LFD-RPA assay is fast, handy, efficient, and suitable for rapid detection of *Salmonella*.

## 2. Materials and Methods

### 2.1. Bacterial Culture and DNA Template Preparation

Eighteen bacterial strains in total were prepared to determine the specificity and sensitivity testing, including two *Salmonella* strains and 16 non-*Salmonella* strains (Table 1). The *Salmonella* standard strain GIMCC1.345 was recovered from −80 °C, and then streaked in *Salmonella* chromogenic agar (Hopebio, China) at 37 °C. After incubation for 16 h, a single colony was picked up in selenite cystine broth (SC, Hopebio). The single colony was grown at 37 °C for 18 h with shaking (200 rpm). After incubating at 37 °C for 16 h, the colony-forming units (CFU) of each plate were determined. Non-*Salmonella* strains were cultured in Luria–Bertani broth (Sangon, China) under the same conditions. Bacterial concentration was determined by plate colony counting. The genomic DNA of *Salmonella* was isolated using a bacterial DNA extraction kit (Sangon, China) according to the manufacturer’s protocol. The DNA concentration was determined by a spectrophotometer (DU730, Beckman Coulter, USA). The purified DNA extractions were stored at −20 °C to evaluate the analytical sensitivity and specificity testing.

When making metagenomic DNA from spiked and actual food samples, 10 mg/mL lysozyme (Bioteke corporation, China) was added to homogenized samples firstly at 37 °C for 5 min, and then 20 mg/mL proteinase K (Bioteke corporation, China) was added at 60 °C for 15 min to help digest proteins in the matrix and lyse the cell wall of the bacteria. The metagenomic DNA was obtained using a bacterial DNA extraction kit (Bioteke corporation, China) according to the manufacturer’s protocol.

### 2.2. Primer Design and Reaction Protocols for Recombinase Polymerase Amplification (RPA)

The primers and probes were manually designed based on the sequence of the conservative segments of the *fimY* gene (GenBank Accession No: JQ665438.1). Based upon the detailed analysis and comparison, primers and probes of RPA were designed using parameters according to the instruction manual (TwistDx) and Primer-BLAST [27] combining Primer 5 and BLAST (Basic Local Alignment Search Tool, National Center for Biotechnology Information) global alignment. All primers (Table 2) were synthesized by Invitrogen Biotechnology Co. Ltd (Shanghai, China).

The purified DNA extractions were used to construct a standard plasmid (the primers listed in Table 2). The PCR primers were used to amplify a 249 bp fragment of the *fimY* gene, which contains RPA primer pairs and a probe-specific binding sequence. Then, the fragment was cloned into the pMD19-T vector to construct a standard plasmid.

The assay was performed with a TwistAmp nfo kit (TwistDX, Cambridge, UK). All reaction mixtures were prepared in a final volume of 50 μL containing 0.4 μM of each primer, 0.1 μM of probe, 14 mM magnesium acetate, lyophilized enzyme pellets, reaction buffer and DNA template. The reaction was incubated at 37 °C for 20 min and then placed on ice. Negative controls (NC) contained nuclease free water instead of DNA. Afterwards, the RPA reactions were quenched by adding 50 μL P/C buffer (trichloromethane and phenol mixed in equal volumes), which removed proteins. The samples were then briefly mixed by flicking and centrifuging at 12,000 rpm for 1 min. The 10 μL supernatant (amplification products) was used for gel electrophoresis. It was performed at 90 V for 30 min. The 2 μL RPA amplification products was mixed with 98 μL of Tris buffer (pH 8.0) to the lateral flow dipsticks and samples migrate by capillary action. The stable incubation time of test strip is 5 min. Finally, the test line intensity was scanned and calculated by the test strip reader.

### 2.3. Optimization of the Lateral Flow Immunoassay with Recombinase Polymerase Amplification (LFD-RPA) Assay

To optimize the assay, different temperature, incubation time, and salt concentrations of magnesium ions were used. The assay was conducted under isothermal conditions between 30 and 50 °C, and the incubation time was extended from 2.5 to 25 min, respectively. In addition, the different concentration of magnesium ions was carried, which ranged from 2.8–16.8 mM. To determine the optimal conditions, 1 μL positive standard plasmid of 10 ng/μL concentration was used as the target template.

### 2.4. Sensitivity and Specificity of the LFD-RPA Assay

The specificity was assessed using DNA extracted from 10^5^ CFU/mL of two *Salmonella* strains and sixteen non-*Salmonella* strains. The sensitivity of the LFD-RPA assay was performed with the extracted DNA of *Salmonella* at concentrations ranging from 1.29 × 10^7^ to 1 CFU/mL. All tests were independently repeated thrice.

### 2.5. Method for Comparison

A TaqMan-based qPCR assay was established, targeting the *fimY* gene. Primers and a probe sequence were designed by Beacon Designer 7.9 (Table 2). The reaction mixture was added in a total volume of 20 µL, and 2.5 µL of each DNA template was added following the monitor by using the instrumentation (IQ^TM^5 optical System, Bio-Rad, USA) for qPCR assay. It contained 10 µL of SensiFAST Probe Lo-ROX (Bioline Inc., London, U.K.), 0.4 µM of primer sets, 0.1 µM of probe and the rest filled with ddH_2_O. The thermal cycle program was described as follows: 95 °C for 2 min, followed by 40 cycles of 95 °C for 10 s and 60 °C for 26 s.

### 2.6. Sample Processing and Artificial Contamination

Tomato, cabbage, and broccoli were obtained from local supermarkets in Hangzhou, China. The samples were verified to be negative for *Salmonella* by bacteriological analytical manual (BAM, Chapter 5, formulated by the Food and Drug Administration), which were used as artificially contaminated samples. Samples (25.0 g ± 0.1 g) were weighed out under sterile conditions with pre-sterilized instruments. To each sample was added 225 mL of universal preenrichment (UP) broth and they were homogenized by using a homogenizer (Bioprep-24, Allsheng Instruments Co. Ltd., Hangzhou, China) at 8000 rpm for 1 min under sterile conditions. They were spiked with *Salmonella* cells with 10^4^, 10^3^, 10^2^, and 10^1^ CFU/mL food homogenate as final concentrations. Immediately, 500 μL of the inoculated samples were collected, and the DNA of each sample was extracted. For the DNA extraction method refer to Section 2.1.

### 2.7. Practical Sample Testing

Actual samples were categorized into two groups based on the source: one group was purchased randomly from the local market, while the others were acquired from the Zhoushan Entry–Exit Inspection and Quarantine Bureau. Each sample (25.0 g ± 0.1 g) was added to 225 mL of buffered peptone water universal (BPW) and mix well by swirling under sterile conditions. Then, it was enriched at 37 °C with shaking at 200 rpm for 16 h after being fully homogenized. After enrichment, 500 μL of each sample for DNA extracting and testing. DNA extraction method refer to Section 2.1. Vegetables samples (tomato, cabbage, and broccoli) were processed according to Section 2.6. All of the samples were subject to by LFD-RPA and qPCR assays, and a traditional method of FDA was utilized in parallel for a comparison.

### 2.8. Statistical Analysis

Data collected from LFD-RPA reactions were analyzed by AS174TestStripReader software (Allsheng Instruments Co. Ltd., Hangzhou, China). Data collected from qPCR reactions including standard curves were analyzed by Bio-Rad iQ5 2.0 software and Microsoft Excel software (Microsoft Inc., USA). Results of qPCR were judged as positive when Cq value ≤35. The standard curve of the LFD-RPA assay was plotted according to T/C value and logarithm of bacterial cultures concentration. Each contamination level of samples was replicated 6 times and each strip scanned by test strip reader three times. The average was used to calculate the recovery rate (the ratio of the actual measured concentration to the concentration of the artificial concentration).

## 3. Results

### 3.1. Assay Principle

**Figure 1:**
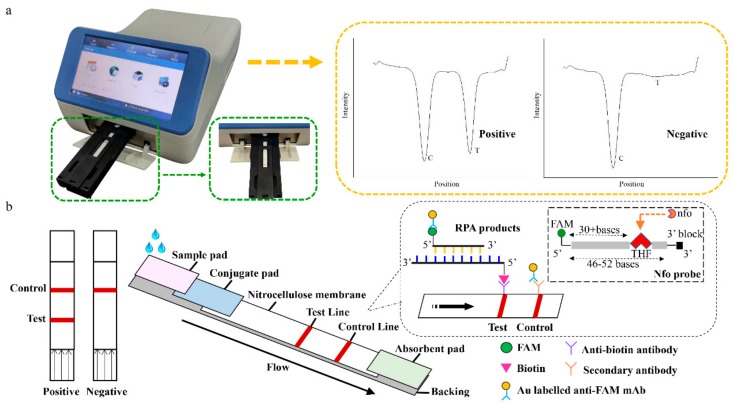
The working principle of the recombinase polymerase amplification combined with lateral flow dipstick (LFD-RPA) assay. (**a**) The test strip reader and the positive/negative test results. (**b**) Schematic diagram of the recombinase polymerase amplification combination with lateral flow dipstick. FAM: carboxyfluorescein, THF: tetrahydrofuran.

The LFD-RPA assay used a pair of specific antibodies to identify the antigen containing two fixed labels for detection. The 5’ end biotin modified primer and the complementary sequences guaranteed the effective amplification of the target binding with the probe. The nfo probe consists of a FAM (carboxyfluorescein) antigenic label at its 5’ end, a tetrahydrofuran (THF) in the middle, and a C3-spacer at the 3’ end. The THF was the abasic-site mimic. The probe’s sequence was homologous to the overlapped region of the primer. It identifies the THF site in the extended double-stranded sequence and shears to form a 3’ hydroxyl substrate catalyzed by polymerase *Bsu* to extend the remaining portion of the probe extending to the remainder of the probe. Finally, amplicons containing biotin and FAM were obtained. The experimental results can be identified by the lateral flow dipstick. The amplification products were run by a capillary force along the nitrocellulose membrane. If the amplification succeeds, a red line is produced on the test wire due to the accumulation of colloidal gold. The uncaptured gold particles flowed through and were immobilized by the second antibody on the control line. In the absence of the target DNA, no red band is observed on the test line (Figure 1b). The entire procedure took approximately 5 min. In addition, the intensity of the test line and control line were scanned by the test strip reader (Figure 1a). The test strip reader converted the received light signals from test (T) line and control (C) line into electrical signals with “T value” and “C value”, and the results were described as the ratio of T to C values (T/C value).

### 3.2. Optimization of the LFD-RPA Conditions

**Figure 2:**
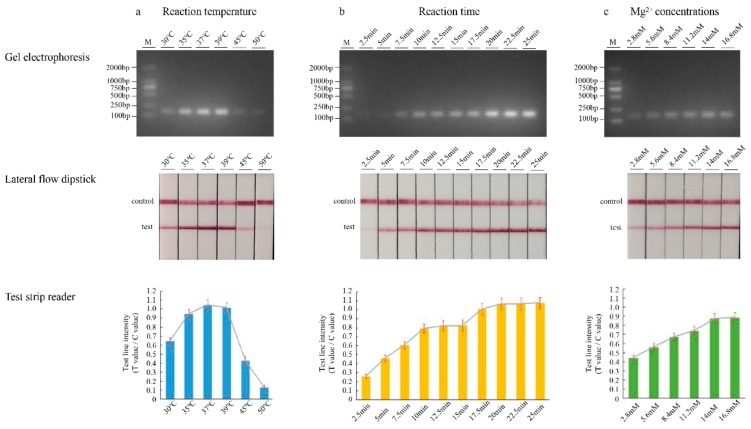
Optimization of the recombinase polymerase amplification reaction temperature (**a**), incubation time (**b**) and the concentration of magnesium ions (**c**) using target DNA. Gel electrophoresis verifying amplification (top), LFD test results (middle), and LFD test line quantification (bottom). Test line intensity was the ratio of the reflected light for the test line and control line.

In order to achieve the optimal amplification effect, we adjusted the amount of pivotal component and reaction parameters in the reaction system. Firstly, six temperature gradients of 30, 35, 37, 39, 45, and 50 °C were conducted to determine the optimal RPA reaction temperature. We assessed this range of temperatures using an incubation time of 20 min and analyzed the amplification products using LF strips. As shown in Figure 2a, strong test lines were obtained at 35, 37, and 39 °C, and no obvious differences were directly observed in the range. When the temperature exceeded 39 °C, as the temperature increased, the stripes gradually became weak. Simultaneously, the test strips were placed into the test strip reader, followed by recording of the intensity of the reflected light for the test line and control line. Consequently, 37 °C was selected as the reaction temperature for subsequent experiments. Next, we tested the effect of incubation time on amplification efficiency. Ten time step gradients of 2.5, 5, 7.5, 10, 12.5, 15, 17.5, 20, 22.5, and 25 min were performed at 37 °C. As the amplification time increased, the brightness of the strip increased. There were no differences between the times of 10 and 15 min (Figure 2b). When the amplification time was 20 or 25 min, the band was brighter, but the reaction time was longer. This result indicates that 10 min is the appropriate reaction time. In addition, both in temperature- and time-optimization experiments, a concentration of 14 mM magnesium ion was used. Subsequently, six concentration gradients of 2.8, 5.6, 8.4, 11.2, 14, and 16.8 mM were tested. We assessed this range of magnesium ion concentrations using an incubation time of 10 min. The luminance of the RPA product analyzed by agarose gel electrophoresis and LFD remained relatively unchanged with concentration, from 14 to 16.8 mM (Figure 2c). Thus, 14 mM magnesium acetate was found to provide an optimal performance for the LFD-RPA assay in this study.

### 3.3. Sensitivity and Specificity

**Figure 3:**
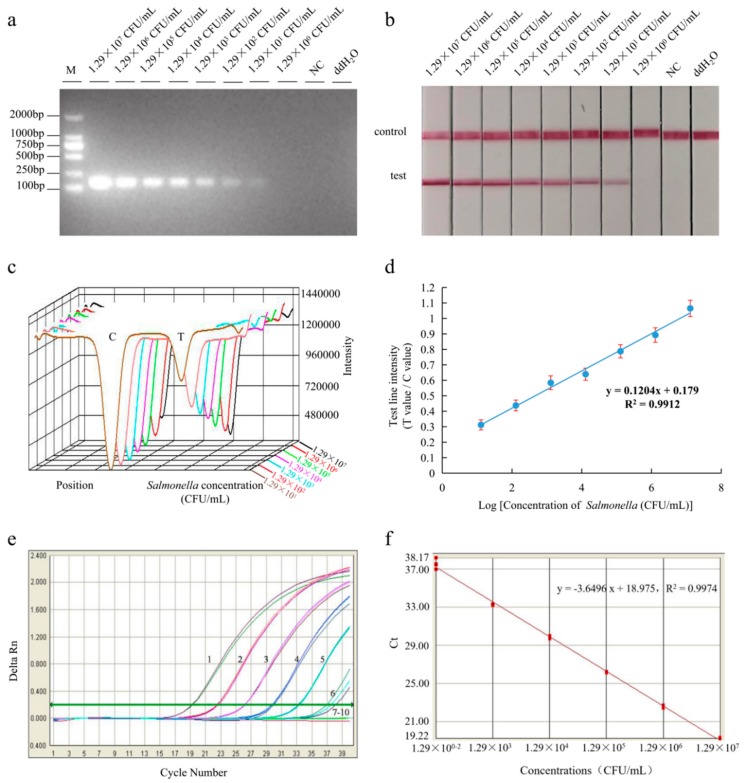
Reaction sensitivity of LFD-RPA and qPCR assays for the *Salmonella* strain culture. The amplified products could be observed with the naked eye by using gel electrophoresis (**a**) and lateral flow dipstick (**b**). The intensity (**c**) was used for quantitative analysis, and it shows a linear correlation (**d**) with the concentration of pure cultures. The qPCR assay (**e**) test concentrations ranged from 1.29 × 10^7^ to 1.29 colony-forming units (CFU)/mL, and the standard curve (**f**) was generated with the Bio-Rad iQ^TM^5 optical System software. 1–8: 1.29 × 10^7^, 1.29 × 10^6^, 1.29 × 10^5^, 1.29 × 10^4^, 1.29 × 10^3^, 1.29 × 10^2^, 1.29 × 10^1^, and 1.29 × 10^0^ CFU/mL; 9: Negative control; 10: ddH_2_O. Test line intensity was the ratio of the reflected light for the test line and control line.

The LFD-RPA assay was applied to pure cultures with different *Salmonella* concentrations ranging from 1.29 × 10^7^ to 1 CFU/mL. The detection sensitivity of the medium was 1.29 × 10^1^ CFU/mL, and the limit of detection of qPCR was 1.29 × 10^2^ CFU/mL (Figure 3).

**Figure 4:**
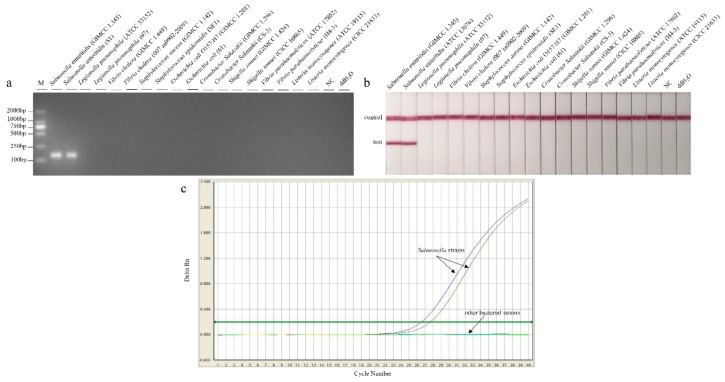
Specificity test of the recombinase polymerase amplification (**a**) combination with lateral flow dipstick (**b**) and qPCR (**c**) assays for targeting *Salmonella*.

As for specificity, the two *Salmonella* strains were identified with a clear visual test line on the strip, whereas the 16 non-*Salmonella* strains only produced a visual band at the control line (Figure 4). No cross-reactions were observed. It revealed that LFD-RPA, based on specific primers, was a reliable assay.

### 3.4. Application to Food Samples

To analyze how different samples affected the LFD-RPA assay, the developed method was evaluated with tomato, cabbage, and broccoli samples spiked with *Salmonella* at concentrations ranging from 10^1^ to 10^4^ CFU/mL. Before artificial contamination, all samples were confirmed to be negative for *Salmonella* using BAM methods. Samples of each produce commodity that were not inoculated were used as negative controls. As shown in Table 3, the recovery of *Salmonella* in the range of (97.13 ± 0.62)% to (102.17 ± 1.32)% were obtained in three spiked samples. The detection limit of *Salmonella* in tomato, cabbage, and broccoli were at the same level. The assay has a slightly higher detection limit for *Salmonella* in food samples than that in pure cultures.

### 3.5. Detection of Practical Products

For practical product testing, 67 different sample types were analyzed (Table 4). *Salmonella* was clearly tested for in all samples using BAM, LFD-RPA, and qPCR assays. The positive detection rates of samples from the local market and Zhoushan Entry–Exit Inspection and Quarantine Bureau were 4% and 0%, respectively.

## 4. Discussion

With the rapid development of foreign trade, national food safety awareness is gradually improving. Food regulators and food producers are facing the same challenges. It is necessary not only to ensure the quality of detection, but also to accelerate the speed and shorten the testing process. The testing of *Salmonella* is very important for finding foodborne pathogens. The detection of *Salmonella* is developing towards the direction of being rapid, simple, of high sensitivity, strong specificity, and low cost. The application of isothermal amplification technology has made microbial detection free from dependence on expensive instruments. It is suitable for field testing and economically underdeveloped areas.

Recently, techniques like polymerase chain reaction (PCR), real-time PCR (qPCR), enzyme-linked immunosorbent assay (ELISA), and gene chip technology for detecting pathogens have developed rapidly and maturely. However, these assays have several drawbacks. Professionals are needed in some steps and the reaction reagents are expensive and unaffordable in rural areas. Therefore, the establishment of rapid and effective detection approaches is crucial. Recombinase polymerase amplification could amplify target fragments in a shorter time (10 min). By contrast, a longer time (40 min) is required for a Loop-mediated isothermal amplification (LAMP) assay, and approximately 40 cycles (40 min) are required in the qPCR assay [1,13,28]. Clearly, the RPA assay is more efficient. The RPA reaction is performed at an ambient constant temperature. Consequently, it evades the need for a high-cost thermo-cycler. In addition, RPA was able to tolerate the impure samples. The tomato, cabbage, and broccoli matrices had little effect on the analytical sensitivity of RPA, and the limit of detection was at a similar level to the pure bacterial culture in our study.

In general, the false-positive results were more likely to occur due to unexpected amplification or primer dimers [14]. An appropriate RPA primer/probe combination could avoid false positives or low sensitivity [29]. Furthermore, studies found that probes played an important role in RPA. In contrast, the change of primers did not have a significant impact [30]. Thus, we referred to the relevant literature in the design process [31]. The nucleotide composition, sequence length, and interplay between primers and probe were carefully considered.

Until now, the utility of isothermal amplification outside the laboratory has been restricted by the lack of signal transduction that is easy-to-use, low cost, and specific [32]. The RPA reaction could be undertaken in a water bath where the temperature is slightly higher than the surrounding temperature [33]. The lateral flow dipstick was free of expensive instruments, and it could be directly observed by the naked eye at room temperature. The LFD-RPA is suitable for testing in the field, and could provide low-cost and instrument-free nucleic acid detection in remote areas. In the previous reports of literatures, LFD-RPA assay was applied to detect *Salmonella* in shellfish with a detection limit of 5 CFU/mL in 8 min at 40 °C [1]. The testing of *Salmonella typhimurium* in milk could successfully reach as low as 1.95 CFU/mL in 10 min at 40–42 °C [29]. Commercially available kits could obtain a reaction result within 15 min at 40 °C [34]. Both were performed with one target fragment in a short time. In this paper, the sensitivity of LFD-RPA assay was 12 CFU/mL within 10 min at 37 °C. Although there is no obvious improvement in terms of reaction time and sensitivity, the artificial reading errors could have been minimaxed by using the reading instrument. The portable TSR-100 test strip reader was not only sensitive, but also could be applied to scan the test strips to decrease the man-made influence and increase the correctness of the diagnosis in this study. By using modern photoelectric technology and a photo-diode to detect the intensity of the reflected light, the reader can calculate the concentration. Such a reader can avoid reading mistakes by eye and perform with rapidity and high sensitivity in order to detect the color change of the test strip. Furthermore, the LFD-RPA assay with the test strip reader has the potential of rapid and quantitative detection in field. However, a few problems need to be solved. To date, LFD cannot distinguish more than two nucleotide amplicons in one RPA reaction system [25]. The challenges for the high-throughput detection of foodborne pathogens will be the focus in future research.

## 5. Conclusions

This study successfully combined recombinase polymerase amplification with a lateral flow immunoassay for rapid detection of *Salmonella* in food. The assay was easy to operate and could be completed in 10 min. It also showed a good specificity and high sensitivity. The reliability was verified by comparing the results of qPCR and BAM. The LFD-RPA method for the detection of *Salmonella* is not only time saving and easy to perform, but also generates specific and sensitive results for routine applications. This method does not require any complex equipment, and can be used as a rapid quantitative detection method with a portable strip reader. The fast and easy-to-read system could be useful as a rapid point-of-care-test for *Salmonella* in food. In summary, the LFD-RPA has practical significance for rapid screening in ports, and detection in grassroots areas or economically underdeveloped areas. However, future essential improvements will reduce potential contamination risks arising when testing many samples in the field.

## Figures and Tables

**Table 1:** Information of bacterial strains used for specificity tests in the study.

Species	ID of Strains	Result of RPA	Result of qPCR
*Salmonella* Enteritidis	GIMCC 1.345	+	+
*Salmonella* Enteritidis	ATCC 13076	+	+
*Legionella pneumophila*	ATCC 33152	−	−
*Legionella pneumophila*	07 *	−	−
*Vibrio cholerae*	GIMCC 1.449	−	−
*Vibrio cholerae*	007 zs0902-2009 *	−	−
*Staphylococcus aureus*	GIMCC 1.142	−	−
*Staphylococcus epidermidis*	SE1 *	−	−
*Escherichia coli* O157:H7	GIMCC 1.201	−	−
*Escherichia coli* O157:H7	61 *	−	−
*Cronobacter Sakazakii*	GIMCC 1.296	−	−
*Cronobacter Sakazakii*	CS-3 *	−	−
*Shigella sonnei*	GIMCC 1.424	−	−
*Shigella flexneri*	CICC 10865	−	−
*Vibrio parahaemolyticus*	ATCC 17802	−	−
*Vibrio parahaemolyticus*	H4-3 *	−	−
*Listeria monocytogenes*	ATCC 19115	−	−
*Listeria monocytogenes*	CICC 21633	−	−

RPA: recombinase polymerase amplification; qPCR: real-time quantitative polymerase chain reaction; GIMCC: Guangdong Microbiology Culture Center, Guangdong, China; ATCC: American Type Culture Collection, Virginia, USA; CICC: China Center of Industrial Culture Collection, Shanghai, China; * Afforded by Zhoushan Entry-exit Inspection and Quarantine Bureau, Zhejiang, China; +: positive result; −: negative result.

**Table 2:** Sequences of *Salmonella* enteritidis primers and probes.

Primer Name	Sequence	Target Gene	Fragment Length
PCR Primers		*fimY*	
Sal-F	5′-CGGTTGGTATCATTTCTC-3’		249 bp
Sal-R	5′-GCAGGTAAATCTGGATATC-3′		
qPCR Primers and Probe			
Sal-qF	5′-CGGTTGGTATCATTTCTC-3′		249 bp
Sal-qR	5′-GCAGGTAAATCTGGATATC-3′		
Sal-qP	5′-FAM-ATTACAACTGACAACTACCTCGGCT-BQH1-3’		
RPA Primers and Probe			
Sal RPA FP1	5′-TATCAGATAAAACCTCCGCTATAACACAGT-3’		131 bp
Sal RPA RP1	5’-Biotin-CTTTCCGATAAGCGAGGTTTGGAGGCTGAT-3’		
Sal RPA P1 nfo Probe	5′-FAM-TCAGATAAAACCTCCGCTATAACACAGTTT[THF]ACGATGGCTGGGCGTT-C3-Spacer		

**Table 3:** Recovery of *Salmonella* in artificially contaminated produce commodities by recombinase polymerase amplification combined with lateral flow dipstick (LFD-RPA) assay.

Samples (n = 6 Each)	Strain	Inoculation Level (CFU/mL)	LFD-RPA Detected Concentration (CFU/mL)	Recovery (%)
tomato	GIMCC1.345	1.29 × 10^4^	+ (1.269 ± 0.014) × 10^4^	98.37 ± 1.09
		1.29 × 10^3^	+ (1.304 ± 0.010) × 10^3^	101.08 ± 0.78
		1.29 × 10^2^	+ (1.253 ± 0.008) × 10^2^	97.13 ± 0.62
		1.29 × 10^1^	−	−
cabbage		1.29 × 10^4^	+ (1.282 ± 0.021) × 10^4^	99.38 ± 1.63
		1.29 × 10^3^	+ (1.264 ± 0.016) × 10^3^	97.98 ± 1.24
		1.29 × 10^2^	+ (1.298 ± 0.011) × 10^2^	100.62 ± 0.85
		1.29 × 10^1^	−	−
broccoli		1.29 × 10^4^	+ (1.312 ± 0.012) × 10^4^	101.71 ± 0.93
		1.29 × 10^3^	+ (1.318 ± 0.017) × 10^3^	102.17 ± 1.32
		1.29 × 10^2^	+ (1.258 ± 0.019) × 10^2^	97.51 ± 1.48
		1.29 × 10^1^	−	−

+, *Salmonella* positive by the method. −, *Salmonella* negative by the method.

**Table 4:** Detection of practical samples by LFD-RPA assay compared with qPCR and bacteriological analytical manual (BAM) method.

Resource	Samples	Number of Samples	Positive Number		
			LFD-RPA	qPCR	BAM
Local market	Shrimp	11	1	1	1
	Tomato	6	0	0	0
	Cabbage	6	0	0	0
	Cod	7	1	1	1
	Meretrix	4	0	0	0
	Broccoli	6	0	0	0
	Chicken	2	0	0	0
	Shellfish	7	0	0	0
	Dried squid	1	0	0	0
	Total	50	2	2	2
	Positive detection rate (%)	/	4.0	4.0	4.0
Zhoushan Entry–Exit Inspection and Quarantine Bureau	Dried squid	17	0	0	0
	Positive detection rate (%)	/	0	0	0
